# Comparison of amyloid deposition in human kidney biopsies as predictor of poor patient outcome

**DOI:** 10.1186/s12882-015-0046-0

**Published:** 2015-04-29

**Authors:** Ekaterina Castano, Matthew B Palmer, Christine Vigneault, Randy Luciano, Serena Wong, Gilbert Moeckel

**Affiliations:** Department of Pathology, Yale University School of Medicine, New Haven, CT USA; Department of Medicine, Yale University School of Medicine, New Haven, CT USA

**Keywords:** Amyloidosis, Kidney, Classification, Outcome, Histology, Biopsy

## Abstract

**Background:**

Amyloidosis leads to deposition of abnormal protein with beta-pleated sheet structure in specific compartments of the affected organs. The histological localization of these amyloid deposits determines the overall survival of the patient.

**Methods:**

In this study we have assessed the histological localization and severity of amyloid deposition in 35 patients with biopsy-proven renal amyloidosis and have compared those to clinical parameters, histo-pathological injury criteria and respective patient outcome. Comparisons were statistically analyzed using thus comparison between the different study groups, which was done using Student *t*-test and analysis of variance.

**Results:**

We find that the glomerulus is by far the most commonly and most severely affected renal compartment and patients with severe glomerular amyloidosis advance faster towards end stage renal disease (ESRD) and death, compared to those patients without glomerular amyloid deposits. Patients with severe glomerular amyloidosis showed higher serum creatinine and urine protein levels, while patients with severe vascular amyloidosis showed higher levels of interstitial inflammatory infiltrate.

**Conclusion:**

In kidneys affected by amyloidosis, the amyloid proteins are predominantly deposited along vessels, especially the small vessels including glomerular capillary loops. The severity of glomerular amyloid deposition enhances the risk of developing ESRD and increases the risk for premature death.

## Background

Renal amyloidosis is caused by deposits of abnormally folded protein with a beta pleated sheet structure in the kidney [1]. These insoluble beta-strands form rope–like amyloid fibrils, which are non-branching and 8-10 nm thick. Such deposits of amyloid protein in vital organs, including the kidney, heart, and muscle, lead to organ dysfunction and are associated with significant morbidity and mortality [2]. Impairment of renal function is a common manifestation of systemic amyloidosis and is often the main cause of morbidity and mortality. Distribution of amyloid differs among patients in regard to organ localization. Different types of amyloid can be seen in renal amyloidosis, but most common are light chain amyloid (AL), associated with multiple myeloma or B cell lymphomas, and acute phase protein-associated amyloid, serum amyloid A (SAA), which can be seen in patients chronic infectious or inflammatory diseases [3]. The annual incidence of AL amyloidosis is estimated at 5-12 cases per million [4], and is the most common form of amyloidosis in developed countries. The amount of circulating light chains may be fairly small and only about 15% of patients with AL amyloidosis fulfill the criteria for multiple myeloma. Significant AA amyloid deposition is dependent on duration of inflammation, magnitude of SAA response and SAA1 homozygosity [5]. AA amyloidosis commonly affects the kidneys and often shows faster progression towards ESRD [2,6].

The correct diagnosis of renal amyloidosis is dependent on the light microscopic findings of eosinophilic amorphous material in glomeruli, vessels or interstitium that is Congo red positive and apple green birefringent upon polarization [7]. Moreover, characteristic immunohistochemistry positivity for kappa or lambda light chains or SAA precursor protein supports the diagnosis for AL or AA amyloidosis respectively [8]. Confirmation of amyloid deposits with non-branching, fibrillary substructure by electron microscopy is essential [9].

Depending on the respective organ affected by amyloidosis, typical clinical symptoms of dysfunction and insufficiency ensue. Impaired organ function due to amyloidosis has been clinically divided into mild, moderate and severe, and has been associated with progressively worse outcome for the patient [10]. The extent and distribution of amyloid deposits in the kidney and the rate of accumulation of new amyloid deposits are important prognostic indicators. The progression of amyloidosis is dependent on the production and concentration of circulating precursor protein, which correlates with deterioration of kidney function [11]. Reduction of SAA serum concentrations in patients with AA amyloidosis improved patient survival [2]. However, an outcome study based on histological localization and amount of amyloid deposits in specific compartments of the kidney has not been performed to date.

In an attempt to standardize pathology reports for renal amyloidosis similar to the lupus nephritis classification, a classification system has been proposed that is based on amyloid deposition in different compartments of the glomerulus [12]. However, this study did not correlate the biopsy classification with clinical outcome. The goal of our study is to evaluate whether the presence of amyloid deposits in a particular renal compartment (glomerular vs. vascular vs. interstitial) may affect patient outcome, including progression to ESRD and death.

## Methods

To identify patients with biopsy proven renal amyloidosis, we conducted a retrospective search of our kidney biopsy repository in the Department of Pathology at Yale University using computerized medical record databases (Copath and EPIC). The Yale University IRB committee approved all patient biopsy material and chart reviews. The biopsies of patients with renal amyloidosis were reviewed by two renal pathologists (GM and MP) and scored for the extent of amyloid deposits in the glomerular, vascular, or interstitial compartments. Review of renal biopsy material included light microscopic evaluation of slides stained with hematoxylin and eosin (H&E), periodic acid Schiff (PAS), Jones silver, Klatskin trichrome, and hematoxylin phloxine saffron (HPS) stains. Congo red stain for amyloid was assessed by light microscopy (LM) for the presence of salmon-colored deposits, which under polarized light showed apple-green birefringence. By electron microscopy (EM), the presence of randomly arrayed non-branching fibrils measuring 8-12 nm in thickness was confirmed. The subtype of amyloid was determined by immunofluorescence or immunohistochemical stains for the expression of Amyloid A, kappa or lambda light chains.

Each patient’s biopsy was evaluated for glomerular, vascular, and interstitial amyloid deposition by assessing the presence and extent of Congo red positive, amorphous, eosinophilic material in blood vessels, glomeruli or interstitial space. To further grade the glomerular amyloid involvement, we developed a scoring system modified from the one published by Sen and Sarcik [12]. We assigned classes of glomerular involvement ranging from 0 to 6. Class 0 biopsies had no glomerular amyloid deposits. Class 1 biopsy cases showed minimal mesangial amyloid deposition (<10% of tuft involved). Class 2 biopsy cases showed mild mesangial amyloid deposition (10-25% of tuft involved). Class 3 biopsy cases showed segmental mesangiocapillary pattern amyloid deposition (26-50% of tuft involved in the mesangial and capillary compartments). Class 4 showed diffuse mesangiocapillary pattern amyloid deposition (51-75% of tuft involved in the mesangial and capillary compartments). Class 5 lesions showed membranous-pattern amyloid deposits in glomeruli (amyloid deposits in the subepithelial compartment). Class 6 lesions showed advanced glomerular amyloid deposition (>76% of tuft involved by amyloid deposits) (Table 1).

**Table 1:** **Amyloid Classification**

Class 0:	No amyloid deposition
Class 1:	Minimal Mesangial (<10%)
Class 2:	Mild Mesangial (10-25%)
Class 3:	Focal mesangiocapillary (26-50%)
Class 4:	Diffuse mesangiocapillary (51-75%)
Class 5:	Membranous
Class 6:	Advanced (>76%)

Class dependent on % of tuft and glomerular compartment involved.

While the scoring system described above is based only on glomerular localization and extend of tuft involvement, we developed an additional scoring system to quantify the amount of amyloid deposition in vascular and interstitial compartments of the renal cortex. We applied a point grid system for the vascular and interstitial areas with amyloid deposits and total points were assigned on a scale from 0 to 3 (0 = absent, 1 = mild (1-25%), 2 = moderate (25-75%), and 3 = severe (75-100%)), where the percentage reflected the area of a vessel or interstitial space filled with amyloid deposits. The final grade for renal amyloidosis in each biopsy was assigned by adding the scores from the two scoring systems described above as follows: grade 1 (summary scores 1-8), grade 2 (summary scores 9-16), and grade 3 (summary scores 17-24) (Table 2). Moreover, scores from 0-3 were also assigned in a similar fashion for the degree of interstitial fibrosis, glomerulosclerosis and interstitial inflammatory infiltrate.

**Table 2:** **Patient Scoring and Grading; Gam = glomerular amyloidosis, GS = glomerulosclerosis, I**_**am**_ **= interstitial amyloidosis, IF = interstitial fibrosis, ILI = interstitial lymphocytic infiltrate, V**_**am**_ **= vascular amyloidosis, Unk = unknown**

**Patient**	**Type**	**Class**	**G**_**am**_	**V**_**am**_	**I**_**am**_	**IF**	**ILI**	**GS**	**Summary score**
1.	AL	4	3	0	1	1	0	0	2(10)
2.	AA	2	1	0	2	1	1	0	1(10)
3.	AA	4	3	3	1	3	2	3	3(19)
4.	AL	6	3	3	1	1	2	2	3(18)
5.	AA	4	3	0	2	1	1	0	2(11)
6.	AL	2	1	0	0	2	1	1	1(17)
7.	AL	2	1	1	0	2	2	0	1(8)
8.	AA	4	3	3	2	2	1	2	3(17)
9.	AL	4	3	0	2	2	1	0	2(12)
10.	AA	2	1	0	0	1	1	0	1(5)
11.	AL	4	2	2	2	3	1	2	2(16)
12.	AL	3	1	1	2	1	1	0	2(9)
13.	AL	6	3	3	2	2	2	3	3(2)
14.	AA	3	2	3	1	1	0	0	2(10)
15.	AL	4	3	2	0	2	2	1	2(14)
16.	AL	6	3	3	1	2	2	2	3(19)
17.	AL	6	3	1	2	1	0	1	2(14)
18.	AL	2	3	0	0	1	1	1	1(8)
19.	AL	4	3	1	1	1	1	1	2(12)
20.	AL	4	3	2	1	1	1	0	2(12)
21	AL	2	3	2	0	1	1	0	2(9)
22.	AL	6	3	1	2	2	1	0	2(15)
23.	AL	0	0	0	1	1	2	0	1(14)
24.	AL	2	3	3	0	1	0	0	2(19)
25.	AL	2	1	0	0	1	1	0	1(15)
26.	AA	4	3	1	1	2	0	0	2(11)
27.	AL	4	3	3	1	2	1	0	2(14)
28.	AA	1	1	3	2	2	2	1	2(12)
29.	AL	1	1	3	1	1	1	0	1(18)
30.	AA	2	3	1	0	2	2	1	2(11)
31.	AL	3	2	1	1	2	2	0	2(11)
32.	AA	6	3	2	2	2	2	1	3(18)
33.	AA	2	1	1	1	1	1		1(18)
34.	AL	1	1	3	1	2	1	2	2(11)
35.	AA	4	3	3	0	2	1	1	2(14)

Furthermore, the EPIC clinical data base was searched for clinical data such as sex, age at diagnosis, clinical presentation, serum creatinine levels at time of biopsy and at time of study, serum albumin levels at time of biopsy and at time of study, level of proteinuria, time interval from diagnosis to ESRD or death, kidney transplant status, documented evidence of systemic amyloidosis, known amyloid sub-type analysis in kidney tissue, patient’s treatment regimen, and years of follow-up. Proteinuria, serum albumin and serum creatinine levels were compared and correlated with degree of amyloidosis.

### Statistical Analysis

All statistical calculations were performed using either the Prism 6 statistical and graphic program or Microsoft Excel version 7 (Microsoft Corporation, NY, USA). Data in the present study was categorical, thus comparison between the different study groups was done using Student *t*-test. All results were reported as a mean ± standard diviation. A probability value (*p* value) less than 0.05 was considered statistically significant, using analysis of variance and Bonferroni t-tests.

## Results

**Figure 1:**
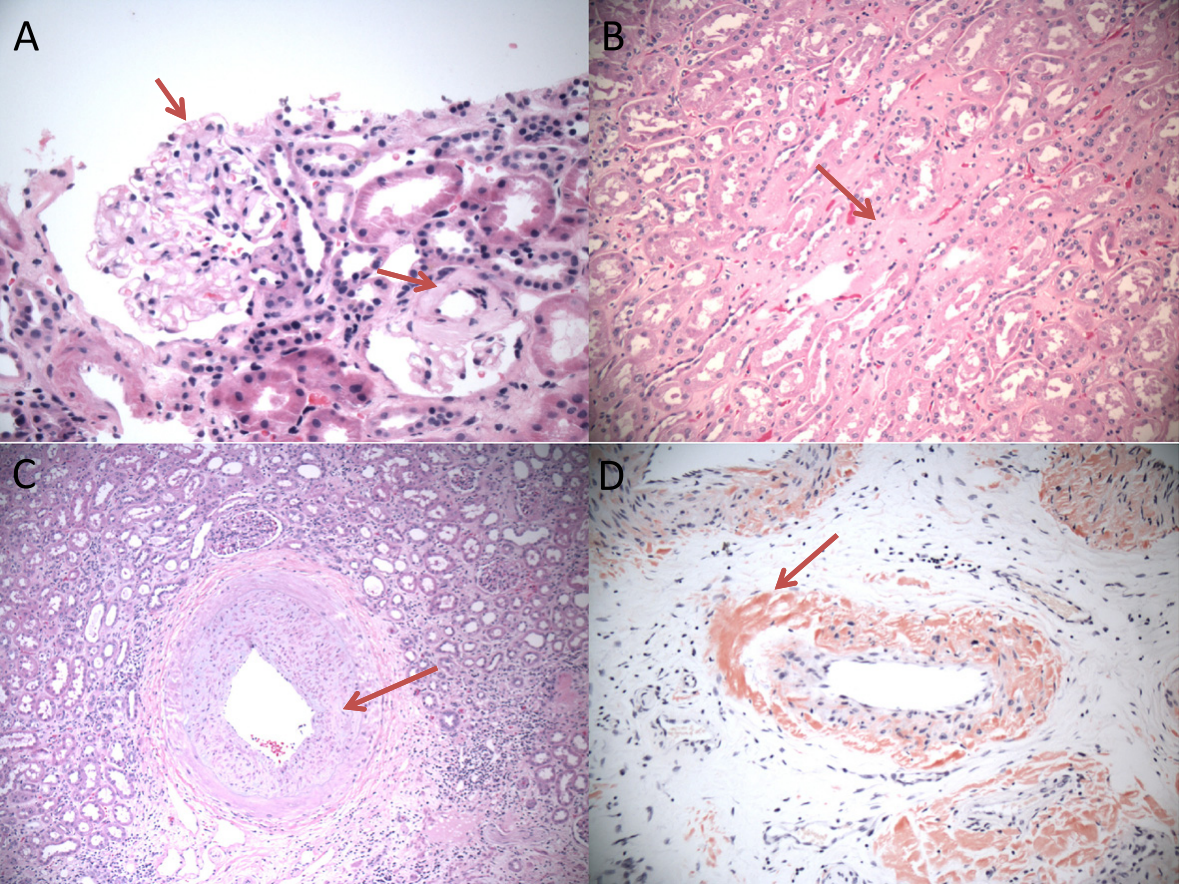
Amyloid deposits in the three major compartments of the kidney: **A**: Glomerular amyloid deposits predominantly in mesangial spaces (400x, red arrow). **B**: Interstitial amyloidosis (200x). **C**: Vascular amyloidosis (100x). **D**: Congo Red stain of vascular amyloid deposits (100x). A-C are H&E stained sections.

**Figure 2:**
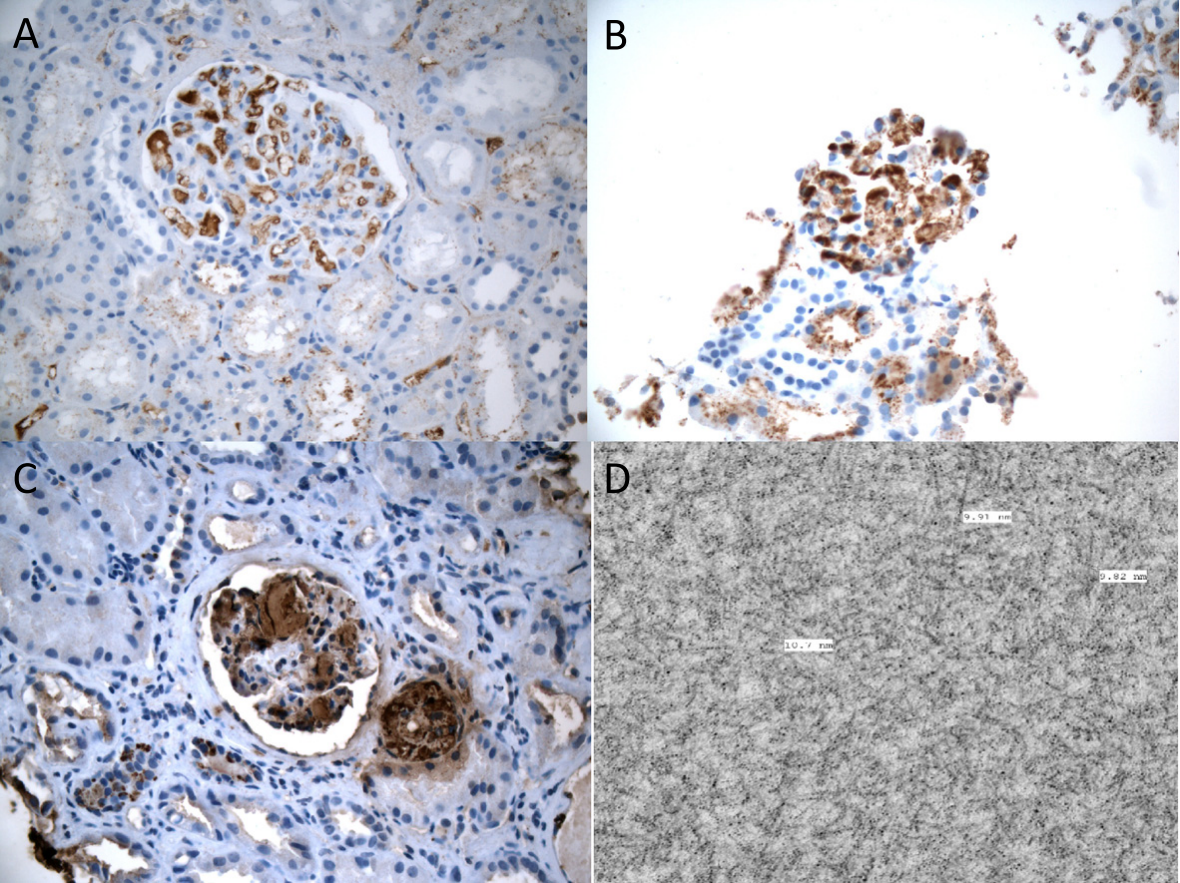
Analysis of AL and AA amyloid with confirmation of ultrastructural characteristics: Immunohistochemistry stains for lambda **(A)** and kappa **(B)** light chains in glomerular amyloid deposits (400x). **C**: Immunohistochemistry stain for AA amyloid in glomerular amyloid deposits (400x). **D**: Electron microscopic image of amyloid deposits showing randomly arranged, non-branching fibrils with thickness ranging between 8 and 10 nm (20,000x).

Our database search resulted in 35 patients with the biopsy diagnosis of renal amyloidosis. Of these, 18 were females and 17 were males. The mean age at diagnosis was 63 years with a range from 37 to 83 years. Review of the biopsy tissue sections of all patients revealed the presence of amyloid, defined by light microscopy as homogeneous eosinophilic material in at least one of the pertinent histological compartments of the kidney, i.e. either media of the arteries or arterioles, mesangium or capillary loops of glomeruli, or the renal interstitium (Figure 1). AL type amyloid was present in 23 patients (66%) and AA type amyloid was seen in 12 patients (34%, Figure 2). Patients with AL amyloidosis were significantly older (mean age 67 years) than those with the AA type (mean age 55 years; p = 0.0015). No sex predilection was found for the type of amyloid (Table 2).

The glomerular tuft was the most common site of amyloid deposition, followed by vascular amyloid. In the kidney biopsy tissues of almost all patients (34 of 35, (97%)), amyloid deposits were found in the glomerular tuft, with either mild or moderate involvement of the vessels and/or the interstitium. However, 11% of patients showed severe glomerular amyloidosis without vascular amyloidosis. Another 20% of biopsies showed mild glomerular amyloidosis and mild to moderate vascular amyloidosis. Only 11% of patients’ biopsies showed mild glomerular amyloidosis but no vascular amyloid deposits (Table 3).

**Table 3:** **Prevalence of Amyloid Deposits in Renal Compartments**

**Renal Compartments with Amyloidosis**	**% Patients with this Localization**	**Average Summary Score of Severity**
Severe Glomerular + severe vascular + mild/mod interstitial	29%	16
Severe Glomerular + mild/mod vascular + mild interstitial	29 %	15
Severe Glomerular + no vascular + mild interstitial	11%	11
Mild Glomerular + mild/mod vascular + mild interstitial	20%	9
Mild Glomerular + no vascular + no interstitial	11%	6

**Figure 3:**
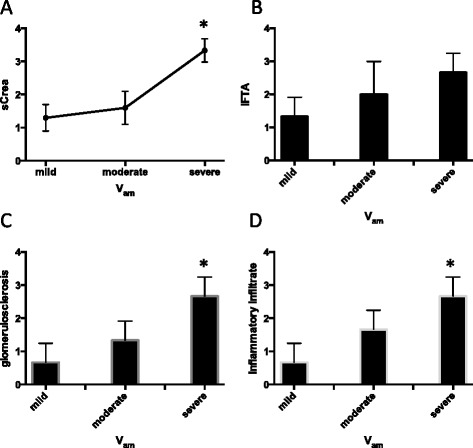
Comparison of serum and tissue parameters in mild, moderate or severe vascular amyloidosis (Vam). **A**: Serum creatinine levels were increased in severe Vam cases compared to mild Vam cases (* = p < 0.05). **B**: The interstitial fibrosis/tubular atrophy (IFTA) grade was increased in severe Vam cases, compared to mild cases. **C**: the grade of glomerulosclerosis was increased in severe Vam cases, compared to mild cases. **D**: the degree of interstitial inflammatory infiltrate increased from mild to moderate to severe vascular amyloidosis grade.

We further assessed the degree of interstitial fibrosis and tubular atrophy, global glomerular sclerosis, and extend of interstitial lymphocytic infiltrate in the kidney biopsies of all 35 patients. The respective fibrosis and glomerular sclerosis scores were correlated with the class and severity of glomerular, vascular, and interstitial amyloid deposition. In patients with vascular amyloidosis the severity of amyloid deposition positively correlated with the degree of glomerular sclerosis (Figure 3C, p = 0.016). Patients with severe vascular amyloidosis also showed higher levels of serum creatinine concentration and interstitial inflammatory infiltrate (Figure 3A and D).

**Figure 4:**
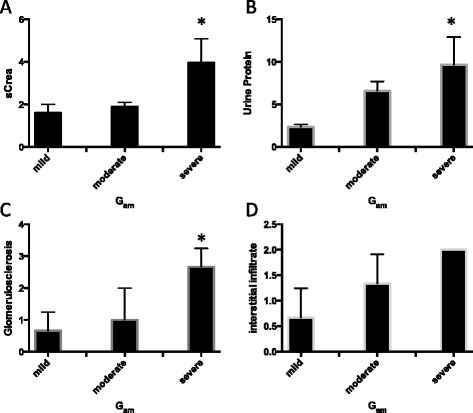
Comparison of serum, urine and tissue parameters in mild, moderate or severe glomerular amyloidosis (Gam). **A**: Serum creatinine levels were significantly increased in severe Gam cases compared to mild Gam cases. **B**: The urine protein levels were significantly higher in severe Gam cases, compared to mild Gam cases. **C**: The grade of glomerulosclerosis was increased in severe Gam cases, compared to mild cases. **D**: The degree of interstitial inflammatory infiltrate increased from mild to moderate to severe Gam cases. * = p < 0.05.

Patients whose kidney biopsies showed severe glomerular amyloidosis also showed the highest levels of serum creatinine concentrations, urine protein concentrations and glomerulosclerosis (Figure 4A, C, D). Patients with severe interstitial amyloidosis were compared to patients with mild interstitial amyloidosis in regard to serum creatinine, serum albumin and urine protein concentrations respectively. The severity of interstitial amyloidosis in kidney biopsies was inversely correlated to the patients’ respective serum albumin levels (p = 0.012).

In order to evaluate the morbidity and mortality associated with the degree of amyloidosis in glomerular, vascular or interstitial compartments, we correlated clinical follow-up data of our patient cohort, such as progression to ESRD, renal replacement therapy (transplantation), or death of the patient with their respective amyloid kidney biopsy classification. Of the 11 patients who progressed to ESRD all showed amyloid deposits in the glomerular tuft. Only one patient received a kidney transplant and subsequently also developed amyloidosis in the allograft. 14 out of 35 patients (40%) had died by the time of study review and all but one showed severe glomerular amyloidosis (93%). Only one of the patients that had died showed mild glomerular and vascular amyloidosis. Of the six patients with mild to moderate glomerular amyloidosis, three progressed to ESRD and one died. Conversely, those patients who had shown minimal glomerular involvement by amyloidosis on biopsy, did not progress to ESRD. When the time span until onset of ESRD or death was compared between patients with predominantly glomerular amyloidosis to those with predominantly interstitial or vascular amyloidosis, no statistical significance could be observed between these patients.

## Discussion

Amyloidosis is a systemic condition that is associated with significant morbidity and mortality and renal involvement with subsequent loss of renal function is one of the factors that determines shortening of life span [10]. In this study we have attempted to assess whether the localization and amount of amyloid in a specific compartment of the kidney tissue is associated with impaired patient outcome. The identification of a certain amyloid deposition pattern indicative of shortened patient survival would be very useful for clinical management at time of biopsy [12]. This study shows that severe glomerular amyloid deposition is indicative of poor patient survival. Moreover, consistent with the literature, our patient cohort showed a higher prevalence for AL amyloidosis than for AA amyloidosis [13]. We found further that the mean age of our patients with AL amyloidosis was 67 years, reflecting the age prevalence for multiple myeloma and plasma cell dyscrasia in the general population [13]. Our study cohort included a high percentage of AA amyloid cases, which most likely reflected the high incidence of chronic inflammation and infection in our patient population.

Almost all biopsies showed amyloid deposits in the glomerular tuft, indicating that the glomerulus is the predominant site of amyloid deposition in the kidney. Eighty percent of biopsies with glomerular amyloid also showed amyloid deposits in small vessels, such as arterioles or interlobular arteries (7/34 do not have vascular →80% do). These findings indicate that renal amyloidosis is a disease of small vessels and glomerular capillaries, which could explain why such a high percentage of patients with severe amyloidosis had either died or had ESRD at the time of our study. Patients with severe vascular amyloidosis also showed the highest levels of inflammatory infiltrate, which indicates that the amyloid deposits throughout the vascular wall might initiate an interstitial inflammation due to closer proximity of vascular deposits to the interstitial capillaries.

Patients with severe glomerular amyloidosis showed the highest levels of serum creatinine concentrations, proteinuria levels, and degree of glomerulosclerosis. These findings indicate that deposition of amyloid in the glomerular tuft significantly reduces glomerular filtration rate, impairs maintenance of the glomerular filtration barrier and leads to subsequent protein loss, which may enhance progression of renal fibrosis. Therefore we conclude that glomerular amyloid deposits are the most important factor influencing the overall loss of renal function in patients with renal amyloidosis.

An important finding of our study is the observation that 93% of the patients of our study cohort who had died, had severe glomerular amyloidosis on kidney biopsy. To the contrary, patients with mild glomerular amyloidosis did not progress towards ESRD. Moreover, only 50% of patients who had moderate glomerular amyloidosis had died. These findings indicate that the extent and amount of amyloid deposition in the glomerular tuft is a predictor of patient mortality. A possible contributing factor may be that severe glomerular amyloid deposition leads to significant proteinuria, which may further accelerate progression of CKD.

## Conclusion

Our study shows that renal involvement with amyloidosis has a predilection for the glomerular compartment and that the extent and amount of amyloid deposits in the glomerular tuft may predict patient outcome.
